# Supplementary material for: Voluntary and involuntary contributions to perceptually guided saccadic choices resolved with millisecond precision
Source: eLife. 2019 Jun 21;8:e46359. doi: 10.7554/eLife.46359 (PMC6645714; doi:10.7554/eLife.46359)
Supplement: Supplementary file 1. [file elife-46359-supp1.pdf]

P/lum	μ_b	σ_b	ρ_b	$\mu_{\mathrm{GO}}^{\mathrm{aff}}$	$\sigma_{\mathrm{GO}}^{\mathrm{aff}}$	$\mu_{\mathrm{CUE}}^{\mathrm{aff}}$	$\sigma_{\mathrm{CUE}}^{\mathrm{aff}}$	μ_{ERI}	σ_{ERI}	g_{ERI}	Δ_{ERI}	a_{EX}	d_{END}	a_{END}	λ
A/high	1.4	3.74	-0.95	51	36	76	5	24	4	0	10	0.96	-0.70	0.17	0.02
A/med	1.4	3.74	-0.95	51	36	104	13	24	3	0	14	1.15	-0.54	0.17	0.02
A/low	1.4	3.74	-0.95	51	36	126	19	24	10	0	14	0.58	-0.29	0.14	0.10
P1/high	4.1	4	-0.92	48	40	76	8	20	2	-0.6	2	0.36	-0.60	0.23	0.00
P1/med	4.1	4	-0.92	48	40	104	15	24	2	-0.5	1	0.39	-0.58	0.22	0.00
P1/low	4.1	4	-0.92	48	40	128	4	9	8	-0.1	3	0.15	-0.20	0.42	0.02
P2/high	4.5	2.37	-0.81	44	34	88	7	16	1	-0.5	6	0.60	-0.90	0.16	0.03
P2/med	4.5	2.37	-0.81	44	34	113	13	17	2	-0.8	8	0.55	-0.80	0.19	0.05
P2/low	4.5	2.37	-0.81	44	34	129	10	11	13	-0.4	7	0.50	-0.64	0.09	0.36
P3/high	1.7	1.7	-0.91	47	40	73	11	26	6	0	15	0.90	-0.59	0.18	0.01
P3/med	1.7	1.7	-0.91	47	40	100	20	22	2	0	17	1.03	-0.18	0.16	0.00
P3/low	1.7	1.7	-0.91	47	40	114	29	22	9	0	10	0.44	-0.27	0.11	0.07
P4/high	2.6	1.34	-0.97	55	41	75	2	14	8	-0.9	5	1.86	-1.05	0.09	0.00
P4/med	2.6	1.34	-0.97	55	41	111	4	17	6	-0.1	4	1.05	-0.72	0.08	0.00
P4/low	2.6	1.34	-0.97	55	41	154	22	16	6	0.0	9	0.76	-0.22	0.04	0.15
P5/high	2.3	1.82	-0.92	54	35	78	6	24	5	-0.4	12	0.81	-0.68	0.11	0.03
P5/med	2.3	1.82	-0.92	54	35	105	5	21	4	-0.2	16	1.00	-0.22	0.11	0.00
P5/low	2.3	1.82	-0.92	54	35	129	19	24	7	-0.4	11	0.43	-0.23	0.10	0.00
P6/high	0.5	1.9	-0.95	46	47	67	8	20	14	-0.6	13	1.50	-1.27	0.13	0.03
P6/med	0.5	1.9	-0.95	46	47	91	7	17	9	-0.3	9	1.36	-0.65	0.11	0.04
P6/low	0.5	1.9	-0.95	46	47	117	12	24	12	-0.1	7	0.62	-0.87	0.13	0.02

Parameters of the race-to-threshold model for individual participants. Participant (P1–P6) and luminance (high, medium, low) are indicated in the first column. For comparison, the results for the aggregate data (A) are shown at the top.
